# Bis(2,4,6-trimethyl­pyridinium) hexa­chloridoplatinate(IV)

**DOI:** 10.1107/S160053680802881X

**Published:** 2008-09-13

**Authors:** Khadijeh Kalateh, Amin Ebadi, Anita Abedi, Vahid Amani, Hamid Reza Khavasi

**Affiliations:** a Islamic Azad University, Shahr-e-Rey Branch, Tehran, Iran; b Department of Chemistry, Islamic Azad University, Kazeroon Branch, Kazeroon, Fars, Iran; c Department of Chemistry, Islamic Azad University, North Tehran Branch, Tehran, Iran; d Department of Chemistry, Shahid Beheshti University, Tehran 1983963113, Iran

## Abstract

The asymmetric unit of the title compound, (C_8_H_12_N)_2_[PtCl_6_], contains one independent protonated 2,4,6-trimethyl­pyridinium cation and one half of a centrosymmetric [PtCl_6_]^2−^ anion. The Pt ion has an almost ideal octa­hedral coordination. In the crystal structure, intra­molecular N—H⋯Cl and inter­molecular C—H⋯Cl hydrogen bonds result in the formation of a supra­molecular structure.

## Related literature

For general background, see: Rafizadeh *et al.* (2006 ▶); Yousefi, Amani & Khavasi (2007 ▶); Abedi *et al.* (2008 ▶); Hojjat Kashani *et al.* (2008 ▶). For related literature, see: Biradha & Zaworotko (1998 ▶); Hallfeldt & Urland (2002 ▶); Foces-Foces *et al.* (1999 ▶); Zordan & Brammer (2004 ▶); Hasan *et al.* (2001 ▶); Juan *et al.* (1998 ▶); Li & Liu (2003 ▶); Hu *et al.* (2003 ▶); Terzis & Mentzafos (1983 ▶); Zordan *et al.* (2005 ▶); Yousefi, Ahmadi *et al.* (2007 ▶); Yousefi *et al.* (2007*a* ▶,*b* ▶); Amani *et al.* (2008 ▶).
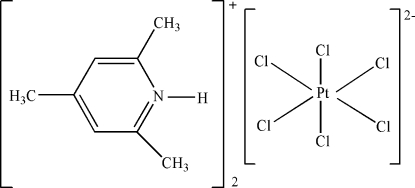


## Experimental

### 

#### Crystal data

(C_8_H_12_N)_2_[PtCl_6_]
*M* *_r_* = 652.15
Triclinic, 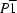

*a* = 7.6302 (8) Å
*b* = 9.1328 (9) Å
*c* = 9.4599 (10) Å
α = 99.201 (8)°
β = 109.683 (8)°
γ = 108.471 (8)°
*V* = 561.87 (12) Å^3^
*Z* = 1
Mo *K*α radiation
μ = 6.96 mm^−1^
*T* = 298 (2) K
0.32 × 0.30 × 0.25 mm

#### Data collection

Bruker SMART CCD area-detector diffractometer
Absorption correction: multi-scan (*SADABS*; Sheldrick, 1998 ▶) *T*_min_ = 0.121, *T*_max_ = 0.176
6510 measured reflections
2962 independent reflections
2952 reflections with *I* > 2σ(*I*)
*R*_int_ = 0.099

#### Refinement

*R*[*F* ^2^ > 2σ(*F* ^2^)] = 0.037
*wR*(*F* ^2^) = 0.088
*S* = 1.18
2962 reflections
116 parameters
H-atom parameters constrained
Δρ_max_ = 1.20 e Å^−3^
Δρ_min_ = −1.44 e Å^−3^

### 

Data collection: *SMART* (Bruker, 1998 ▶); cell refinement: *SAINT* (Bruker, 1998 ▶); data reduction: *SAINT*; program(s) used to solve structure: *SHELXTL* (Sheldrick, 2008 ▶); program(s) used to refine structure: *SHELXTL*; molecular graphics: *ORTEP-3 for Windows* (Farrugia, 1997 ▶); software used to prepare material for publication: *WinGX* (Farrugia, 1999 ▶).

## Supplementary Material

Crystal structure: contains datablocks I, global. DOI: 10.1107/S160053680802881X/hk2526sup1.cif

Structure factors: contains datablocks I. DOI: 10.1107/S160053680802881X/hk2526Isup2.hkl

Additional supplementary materials:  crystallographic information; 3D view; checkCIF report

## Figures and Tables

**Table:** 

Pt1—Cl1	2.3225 (11)
Pt1—Cl2	2.3199 (12)
Pt1—Cl3	2.3197 (13)

**Table:** 

Cl2—Pt1—Cl1^i^	89.35 (5)
Cl2—Pt1—Cl1	90.65 (5)
Cl3—Pt1—Cl1	90.10 (5)
Cl3—Pt1—Cl1^i^	89.90 (5)
Cl3—Pt1—Cl2	90.45 (6)
Cl3—Pt1—Cl2^i^	89.55 (6)

Symmetry code: (i) 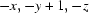.

**Table 2:** Hydrogen-bond geometry (Å, °)

*D*—H⋯*A*	*D*—H	H⋯*A*	*D*⋯*A*	*D*—H⋯*A*
N1—H1*D*⋯Cl2	0.86	2.45	3.301 (5)	173
C1—H1*C*⋯Cl1^ii^	0.96	2.81	3.743 (6)	165
C8—H8*A*⋯Cl3^iii^	0.96	2.80	3.731 (10)	163

Symmetry codes: (ii) 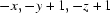; (iii) 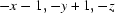.

**Fig. 1.**
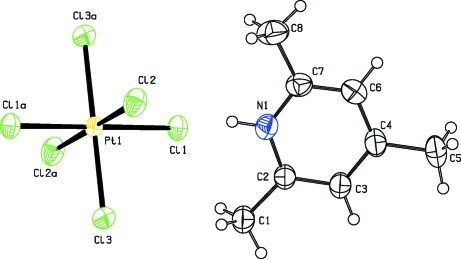
The molecular structure of the title molecule with the atom-numbering scheme. Displacement ellipsoids are drawn at the 40% probability level [symmetry code: (a) x, y + 1, z].

**Fig. 2.**
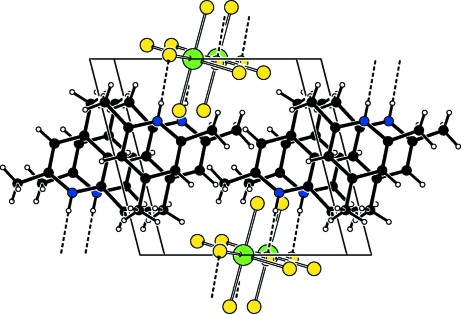
A packing diagram of the title compound. Hydrogen bonds are shown as dashed lines.

## supplementary crystallographic information

### Comment

In recent years, there has been considerable interest in proton transfer systems and their structures (Rafizadeh *et al.*,  2006; Yousefi, Amani & Khavasi, 2007; Abedi *et al.*,  2008; Hojjat Kashani *et al.*, 2008). Several proton transfer systems using 2,4,6-trimethylpyridine, with proton donor molecules, such as [2,4,6-tmpy.H]_2_[H_2_BTEC], (II), (Biradha & Zaworotko, 1998), {[2,4,6-tmpy.H]_10_[Er(H_2_O)Cl_5_]_2_[ErCl_6_]3Cl}, (III), (Hallfeldt & Urland, 2002), [2,4,6-tmpy.H][2-NBA], (IV) and [2,4,6-tmpy.H][3,5-NBA], (V), (Foces-Foces *et al.*,  1999) [where 2,4,6-tmpy.H is 2,4,6-trimethylpyridinium, H_2_BTEC is dihydrogen-1,2,4,5-benzenetetracarboxylate, 2-NBA is 2-nitrobenzoate and 3,5-NBA is 3,5- nitrobenzoate] have been synthesized and characterized by single-crystal X-ray diffraction methods.

There are also several proton transfer systems using H_2_[PtCl_6_] with proton acceptor molecules, such as [HpyBr-3]_2_[PtCl_6_].2H_2_O, (VI), and [HpyI-3]_2_[PtCl_6_].2H_2_O, (VII),(Zordan & Brammer, 2004), [BMIM]_2_[PtCl_6_], (VIII), and [EMIM]_2_[PtCl_6_], (IX), (Hasan *et al.*,  2001), {(DABCO)H_2_[PtCl_6_]}, (*X*), (Juan *et al.*,  1998), {*p*-C_6_H_4_(CH_2_ImMe)_2_[PtCl_6_]}, (XI), (Li & Liu, 2003), [het][PtCl_6_].2H_2_O, (XII), (Hu *et al.*,  2003), [9-MeGuaH]_2_[PtCl_6_].2H_2_O, (XIII), (Terzis & Mentzafos, 1983), [HpyCl-3]_3_[PtCl_6_]Cl, (XIV), (Zordan *et al.*,  2005), [2,9-dmphen.H]_2_[PtCl_6_], (XV), (Yousefi, Ahmadi *et al.*, 2007), [H_2_DA18C6][PtCl_6_].2H_2_O, (XVI), (Yousefi *et al.*, 2007a), [2,6-dmpy.H]_2_[PtCl_6_], (XVII), (Amani *et al.*,  2008) and [TBA]_3_[PtCl_6_]Cl, (XVIII), (Yousefi *et al.*, 2007b) [where hpy is halo-pyridinium, BMI*M*^+^ is 1-*n*-butyl-3-methylimidazolium, EMI*M*^+^ is 1-ethyl-3-methyl- imidazolium, DABCO is 1,4-diazabicyclooctane, Im is imidazolium, het is 2-(α-hydroxyethyl)thiamine, 9-MeGuaH is 9-methylguaninium, 2,9-dmphen.H is 2,9-dimethyl-1,10-phenanthrolinium, H_2_DA18C6 is 1,10-Diazonia-18-crown-6, 2,6-dmpy.H is 2,6-dimethylpyridinium and TBA is tribenzylammonium] have been synthesized and characterized by single-crystal X-ray diffraction methods. We report herein the synthesis and crystal structure of the title compound, (I).

The asymmetric unit of (I), (Fig. 1) contains one independent protonated 2,4,6-trimethylpyridinium cation and one half of a centrosymmetric [PtCl_6_]^2-^ anion. The Pt ion has an octahedral coordination. In cation, the bond lengths and angles are in good agreement with the corresponding values in (II) and (IV). In [PtCl_6_]^2-^ anion, the Pt—Cl bond lengths and Cl—Pt—Cl bond angles (Table 1) are also within normal ranges, as in (XVI), (XVII) and (XVIII).

In the crystal structure (Fig. 2), intramolecular N—H···Cl and intermolecular C—H···Cl hydrogen bonds (Table 2) result in the formation of a supramolecular structure, in which they may be effective in the stabilization of the structure.

### Experimental

For the preparation of the title compound, (I), a solution of 2,4,6-trimethylpyridine (0.18 g, 1.48 mmol) in methanol (15 ml) was added to a solution of H_2_PtCl_6_.6H_2_O, (0.38 g, 0.74 mmol) in acetonitrile (25 ml) and the resulting yellow solution was stirred for 10 min at 313 K. Then, it was left to evaporate slowly at room temperature. After one week, orange prismatic crystals of (I) were isolated (yield; 0.35 g; 72.5%).

### Refinement

H atoms were positioned geometrically, with N—H = 0.86 Å (for NH) and C—H = 0.93 and 0.96 Å for aromatic and methyl H, respectively, and constrained to ride on their parent atoms with U_iso_(H) = 1.2U_eq_(C,N).

### Crystal data

**Table:**

(C_8_H_12_N)_2_[PtCl_6_]	*Z* = 1
*M**_r_* = 652.15	*F*(000) = 314
Triclinic, *P*1	*D*_x_ = 1.927 Mg m^−^^3^
Hall symbol: -P 1	Mo *K*α radiation, λ = 0.71073 Å
*a* = 7.6302 (8) Å	Cell parameters from 1715 reflections
*b* = 9.1328 (9) Å	θ = 3.0–29.1°
*c* = 9.4599 (10) Å	µ = 6.96 mm^−^^1^
α = 99.201 (8)°	*T* = 298 K
β = 109.683 (8)°	Prism, orange
γ = 108.471 (8)°	0.32 × 0.30 × 0.25 mm
*V* = 561.87 (12) Å^3^	

### Data collection

**Table:**

Bruker SMART CCD area-detector diffractometer	2962 independent reflections
Radiation source: fine-focus sealed tube	2952 reflections with *I* > 2σ(*I*)
graphite	*R*_int_ = 0.099
φ and ω scans	θ_max_ = 29.1°, θ_min_ = 3.0°
Absorption correction: multi-scan (SADABS; Sheldrick, 1998)	*h* = −10→10
*T*_min_ = 0.121, *T*_max_ = 0.176	*k* = −12→12
6510 measured reflections	*l* = −12→12

### Refinement

**Table:**

Refinement on *F*^2^	Secondary atom site location: difference Fourier map
Least-squares matrix: full	Hydrogen site location: inferred from neighbouring sites
*R*[*F*^2^ > 2σ(*F*^2^)] = 0.037	H-atom parameters constrained
*wR*(*F*^2^) = 0.088	*w* = 1/[σ^2^(*F*_o_^2^) + (0.0557*P*)^2^] where *P* = (*F*_o_^2^ + 2*F*_c_^2^)/3
*S* = 1.18	(Δ/σ)_max_ = 0.010
2962 reflections	Δρ_max_ = 1.20 e Å^−^^3^
116 parameters	Δρ_min_ = −1.44 e Å^−^^3^
0 restraints	Extinction correction: SHELXTL (Sheldrick, 1998), Fc^*^=kFc[1+0.001xFc^2^λ^3^/sin(2θ)]^-1/4^
Primary atom site location: structure-invariant direct methods	Extinction coefficient: 0.115 (6)

### Special details

**Table:**

Geometry. All e.s.d.'s (except the e.s.d. in the dihedral angle between two l.s. planes) are estimated using the full covariance matrix. The cell e.s.d.'s are taken into account individually in the estimation of e.s.d.'s in distances, angles and torsion angles; correlations between e.s.d.'s in cell parameters are only used when they are defined by crystal symmetry. An approximate (isotropic) treatment of cell e.s.d.'s is used for estimating e.s.d.'s involving l.s. planes.
Refinement. Refinement of *F*^2^ against ALL reflections. The weighted *R*-factor *wR* and goodness of fit *S* are based on *F*^2^, conventional *R*-factors *R* are based on *F*, with *F* set to zero for negative *F*^2^. The threshold expression of *F*^2^ > σ(*F*^2^) is used only for calculating *R*-factors(gt) *etc*. and is not relevant to the choice of reflections for refinement. *R*-factors based on *F*^2^ are statistically about twice as large as those based on *F*, and *R*- factors based on ALL data will be even larger.

### Fractional atomic coordinates and isotropic or equivalent isotropic displacement parameters (Å^2^)

**Table:**

	*x*	*y*	*z*	*U*_iso_*/*U*_eq_	
Pt1	0.0000	0.5000	0.0000	0.03163 (12)	
Cl1	0.1751 (2)	0.65180 (16)	0.26337 (11)	0.0496 (3)	
Cl2	−0.2671 (2)	0.5772 (2)	−0.01978 (15)	0.0522 (3)	
Cl3	−0.1501 (2)	0.27660 (16)	0.07023 (13)	0.0486 (3)	
N1	−0.2844 (7)	0.7163 (6)	0.3174 (5)	0.0473 (9)	
H1D	−0.2918	0.6752	0.2261	0.057*	
C1	−0.2767 (12)	0.4641 (8)	0.3642 (6)	0.0567 (14)	
H1A	−0.1645	0.4737	0.3364	0.068*	
H1B	−0.4017	0.3993	0.2738	0.068*	
H1C	−0.2670	0.4137	0.4465	0.068*	
C2	−0.2721 (8)	0.6278 (7)	0.4197 (5)	0.0448 (10)	
C3	−0.2540 (10)	0.6948 (7)	0.5686 (6)	0.0496 (11)	
H3	−0.2448	0.6366	0.6410	0.059*	
C4	−0.2496 (10)	0.8492 (8)	0.6091 (6)	0.0533 (12)	
C5	−0.2300 (17)	0.9231 (11)	0.7709 (8)	0.077 (2)	
H5A	−0.3464	0.9469	0.7620	0.092*	
H5B	−0.1094	1.0211	0.8227	0.092*	
H5C	−0.2215	0.8483	0.8310	0.092*	
C6	−0.2651 (10)	0.9340 (7)	0.4965 (7)	0.0540 (12)	
H6	−0.2611	1.0380	0.5221	0.065*	
C7	−0.2858 (9)	0.8639 (7)	0.3499 (6)	0.0493 (11)	
C8	−0.3106 (14)	0.9436 (10)	0.2211 (9)	0.0687 (18)	
H8A	−0.4370	0.8775	0.1325	0.082*	
H8B	−0.2007	0.9562	0.1898	0.082*	
H8C	−0.3100	1.0478	0.2589	0.082*	

### Atomic displacement parameters (Å^2^)

**Table:**

	*U*^11^	*U*^22^	*U*^33^	*U*^12^	*U*^13^	*U*^23^
Pt1	0.03766 (15)	0.03245 (15)	0.02614 (14)	0.01452 (9)	0.01484 (8)	0.00745 (7)
Cl1	0.0642 (7)	0.0451 (6)	0.0288 (4)	0.0166 (5)	0.0150 (4)	0.0042 (3)
Cl2	0.0546 (6)	0.0726 (8)	0.0457 (5)	0.0397 (6)	0.0252 (5)	0.0200 (5)
Cl3	0.0621 (7)	0.0406 (6)	0.0440 (5)	0.0148 (5)	0.0274 (5)	0.0148 (4)
N1	0.049 (2)	0.052 (2)	0.0380 (16)	0.0178 (18)	0.0172 (15)	0.0113 (15)
C1	0.076 (4)	0.061 (3)	0.045 (2)	0.039 (3)	0.028 (2)	0.016 (2)
C2	0.052 (2)	0.047 (2)	0.0385 (18)	0.024 (2)	0.0190 (17)	0.0110 (16)
C3	0.062 (3)	0.052 (3)	0.041 (2)	0.028 (2)	0.0239 (19)	0.0121 (18)
C4	0.062 (3)	0.054 (3)	0.044 (2)	0.026 (2)	0.023 (2)	0.0060 (19)
C5	0.116 (7)	0.067 (5)	0.052 (3)	0.044 (5)	0.039 (4)	0.005 (3)
C6	0.059 (3)	0.042 (3)	0.057 (3)	0.018 (2)	0.023 (2)	0.012 (2)
C7	0.046 (2)	0.047 (3)	0.049 (2)	0.0134 (19)	0.0164 (18)	0.0172 (19)
C8	0.074 (4)	0.063 (4)	0.067 (3)	0.025 (3)	0.024 (3)	0.033 (3)

### Geometric parameters (Å, °)

**Table:**

Pt1—Cl1	2.3225 (11)	C3—H3	0.9300
Pt1—Cl1^i^	2.3225 (11)	C4—C6	1.409 (9)
Pt1—Cl2	2.3199 (12)	C4—C5	1.507 (8)
Pt1—Cl2^i^	2.3199 (12)	C5—H5A	0.9600
Pt1—Cl3^i^	2.3197 (13)	C5—H5B	0.9600
Pt1—Cl3	2.3197 (13)	C5—H5C	0.9600
N1—H1D	0.8600	C6—C7	1.365 (9)
C1—C2	1.488 (8)	C6—H6	0.9300
C1—H1A	0.9600	C7—N1	1.338 (8)
C1—H1B	0.9600	C7—C8	1.505 (8)
C1—H1C	0.9600	C8—H8A	0.9600
C2—N1	1.355 (7)	C8—H8B	0.9600
C2—C3	1.385 (6)	C8—H8C	0.9600
C3—C4	1.388 (8)		
			
Cl1—Pt1—Cl1^i^	180.0	C2—C3—C4	119.7 (5)
Cl2—Pt1—Cl1^i^	89.35 (5)	C2—C3—H3	120.2
Cl2^i^—Pt1—Cl1^i^	90.65 (5)	C4—C3—H3	120.2
Cl2—Pt1—Cl1	90.65 (5)	C3—C4—C6	118.9 (5)
Cl2—Pt1—Cl2^i^	180.0	C3—C4—C5	120.0 (6)
Cl3—Pt1—Cl1	90.10 (5)	C6—C4—C5	121.1 (6)
Cl3^i^—Pt1—Cl1^i^	90.10 (5)	C4—C5—H5A	109.5
Cl3—Pt1—Cl1^i^	89.90 (5)	C4—C5—H5B	109.5
Cl3—Pt1—Cl2	90.45 (6)	H5A—C5—H5B	109.5
Cl3^i^—Pt1—Cl2^i^	90.45 (6)	C4—C5—H5C	109.5
Cl3—Pt1—Cl2^i^	89.55 (6)	H5A—C5—H5C	109.5
Cl3^i^—Pt1—Cl3	180.0	H5B—C5—H5C	109.5
C2—N1—H1D	118.0	C7—C6—C4	120.1 (5)
C7—N1—C2	123.9 (5)	C7—C6—H6	119.9
C7—N1—H1D	118.0	C4—C6—H6	119.9
C2—C1—H1A	109.5	N1—C7—C6	118.9 (5)
C2—C1—H1B	109.5	N1—C7—C8	117.6 (6)
H1A—C1—H1B	109.5	C6—C7—C8	123.5 (6)
C2—C1—H1C	109.5	C7—C8—H8A	109.5
H1A—C1—H1C	109.5	C7—C8—H8B	109.5
H1B—C1—H1C	109.5	H8A—C8—H8B	109.5
N1—C2—C3	118.5 (5)	C7—C8—H8C	109.5
N1—C2—C1	117.3 (4)	H8A—C8—H8C	109.5
C3—C2—C1	124.2 (5)	H8B—C8—H8C	109.5
			
C1—C2—N1—C7	178.8 (6)	C3—C4—C6—C7	0.6 (10)
C3—C2—N1—C7	−1.9 (9)	C5—C4—C6—C7	−179.0 (7)
N1—C2—C3—C4	0.2 (9)	C4—C6—C7—N1	−2.2 (10)
C1—C2—C3—C4	179.5 (6)	C4—C6—C7—C8	177.8 (7)
C2—C3—C4—C6	0.4 (10)	C6—C7—N1—C2	2.9 (9)
C2—C3—C4—C5	180.0 (7)	C8—C7—N1—C2	−177.0 (6)

Symmetry codes: (i) −*x*, −*y*+1, −*z*.

### Hydrogen-bond geometry (Å, °)

**Table:**

*D*—H···*A*	*D*—H	H···*A*	*D*···*A*	*D*—H···*A*
N1—H1D···Cl2	0.86	2.45	3.301 (5)	173.
C1—H1C···Cl1^ii^	0.96	2.81	3.743 (6)	165.
C8—H8A···Cl3^iii^	0.96	2.80	3.731 (10)	163.

Symmetry codes: (ii) −*x*, −*y*+1, −*z*+1; (iii) −*x*−1, −*y*+1, −*z*.
